# Supplementary material for: Upregulation of Neuropilin-1 Inhibits HTLV-1 Infection
Source: Pathogens. 2023 Jun 15;12(6):831. doi: 10.3390/pathogens12060831 (PMC10305173; doi:10.3390/pathogens12060831)
Supplement: Supplementary file 1 [file pathogens-12-00831-s001.zip › pathogens-2430104-supplementary.pdf]

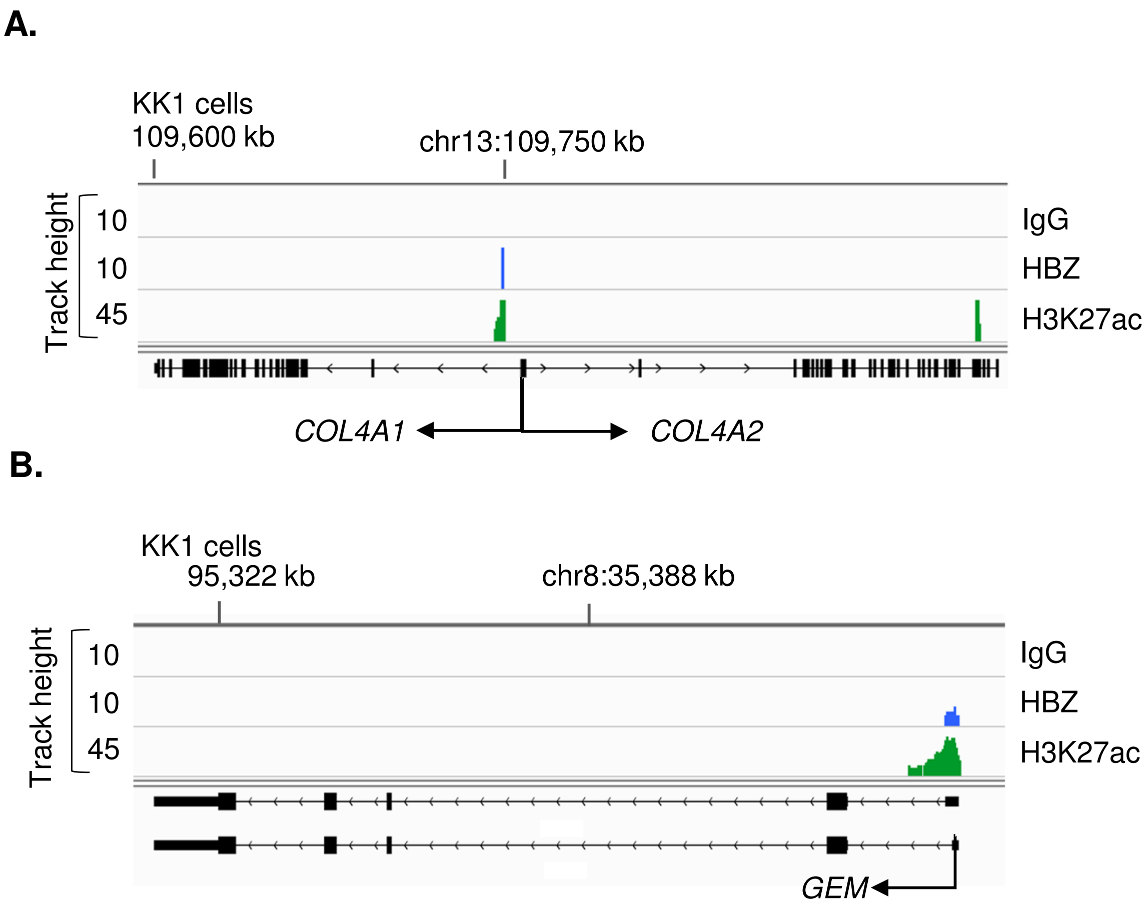

Figure S1. HBZ activates COL4A1, COL4A2 and GEM expression in ATL-derived cell lines. HBZ associates with chromosomal sites near the (**A**) COL4A1/COL4A2 and (**B**) GEM transcription start sites (indicated by the arrows). ChIP-seq tracks for HBZ, H3K27ac, and negative control IgG are shown across the NRP1 locus in KK1 cells using the IGV Browser. Genomic coordinates are based on the NCBI36/hg18 assembly. Data were obtained from published Data sets (GEO accession number GSE94732; [52]). Changes in (**C**) COL4A1, (**D**) COL4A2 and (**E**) GEM expression following deletion of HBZ in ST1 and KK1 ATL-derived cell lines. Graphs were generated from published microarray data (GEO accession number GSE94409; [52]) and show transcript levels after inducing CRISPR/Cas9-mediated knockout of HBZ, using two different guide RNAs (sgHBZ_1 and _2). Data are from day 8 post-induction except for sgHBZ_2 in KK1, which is the day 7 values (no day 8 data provided for this specimen). Values were obtained using GEO2R with calculations based on averaged values from the two array features for COL4A1 and the single features for COL4A2 and GEM.

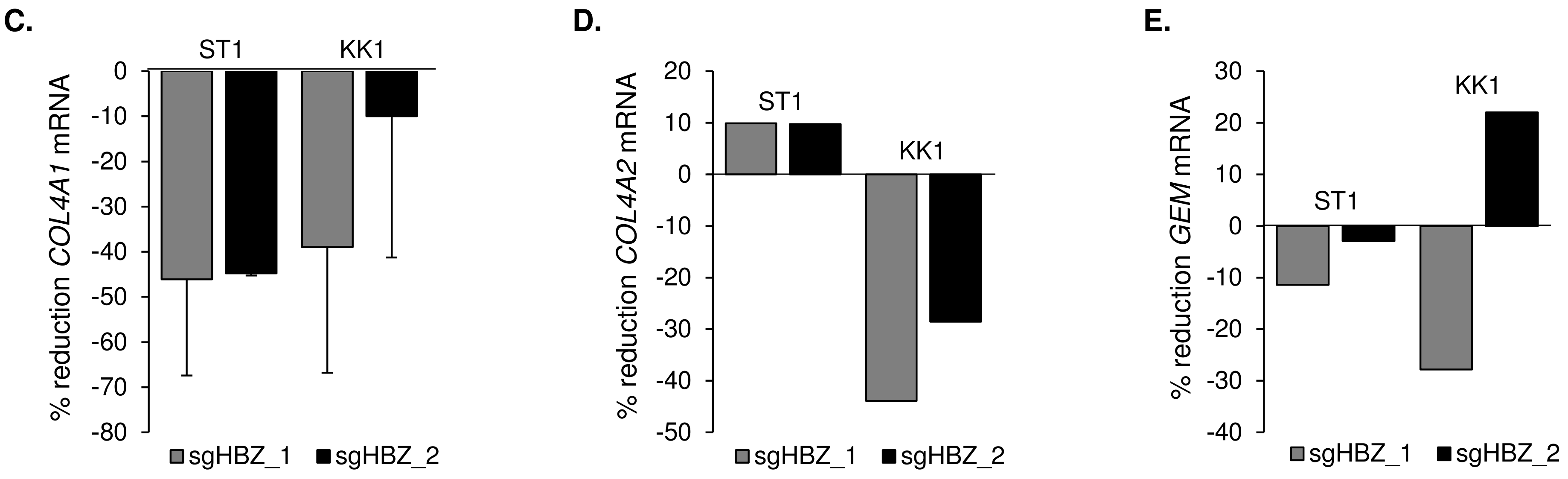

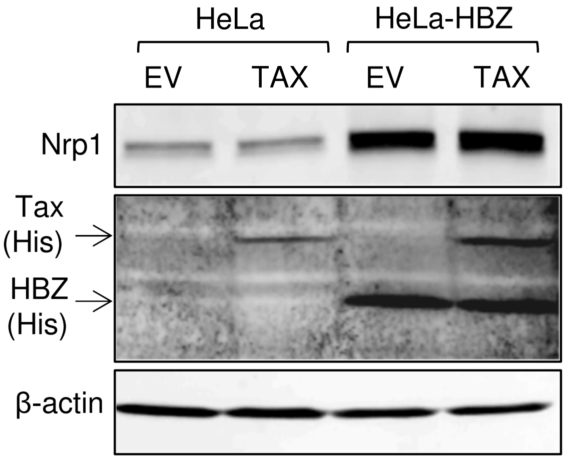

Figure S2. Nrp1 expression in Tax transfected HeLa cells. HeLa cells were transfected with 4 µg of empty vector (pSG5) or pSG-Tax-6His for 48h. Whole cell extracts (50 µg) were analyzed by western blot using antibodies against Nrp1, HBZ (6xHis epitope) and β-actin.

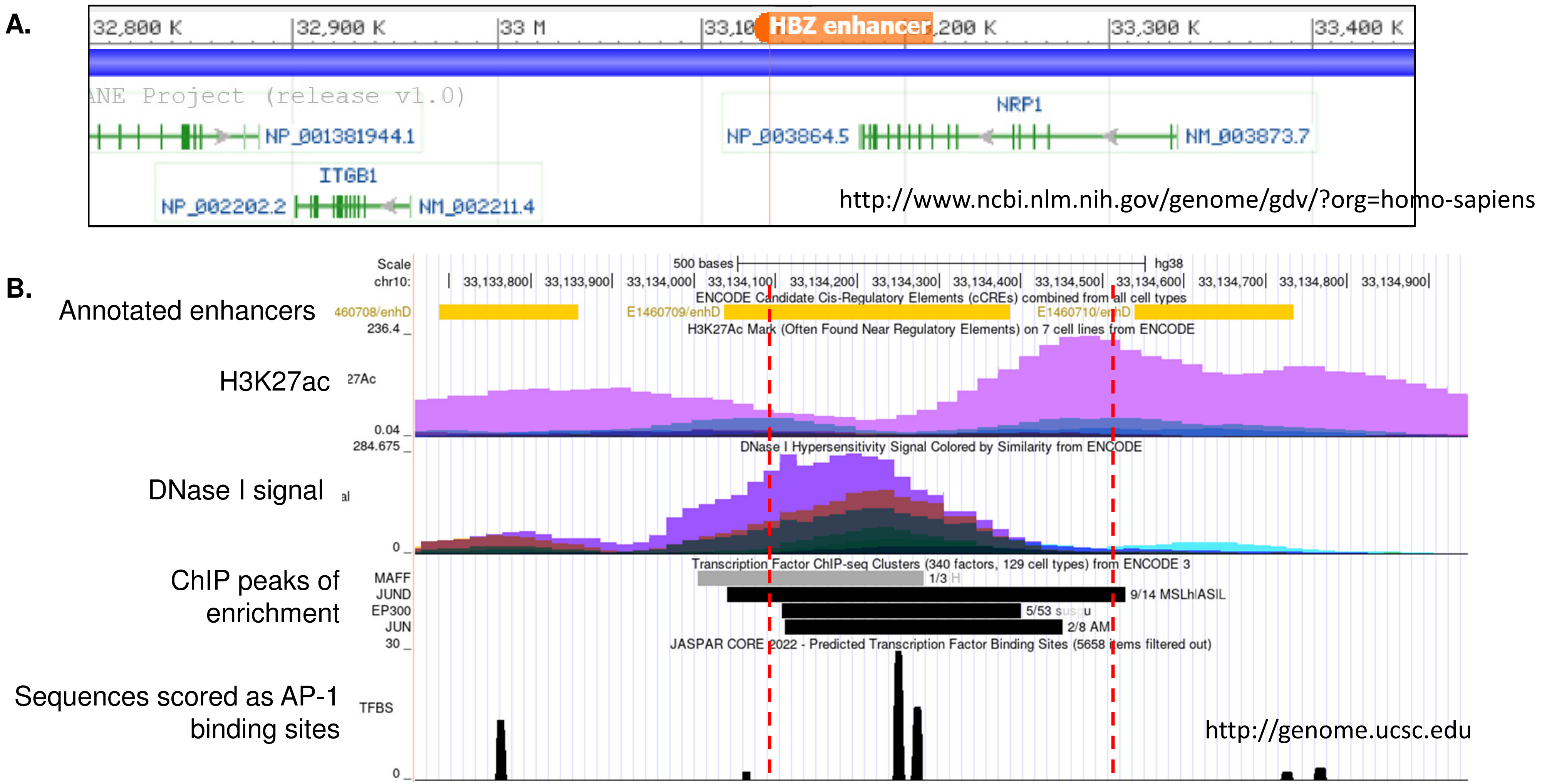

C.

CCCCAGGTGGTAAAATTGTTATCCACCTTCGTCACCTCTTTCCTAATGGAGGAAGTGAGGACAGGCAGCCTTGGAGTCCTACTTGAATG
AGGCCTGGACCTTATGCAGGGTAATAAAAACCAACACGTGGGGCCTGTGCTGTAAGGTGGCAGAGGGCGA**TGACTCA**CCCCACTCATGC
AGGT**TGAGTCA**TTTCAAGGCAGAGTTGTGCCAGTTCAGTACCCAGTAATATTTTCCAGTCGACGAGTATCAGTGAACAGGAGATAACCA
GTCATTTCTAGATTCTGCTCAGAGTCCCAGCTTAGAGGCTCCACCAGCTCAAAGAGACGGGATGGCAAAACAGCCACCTTAATTTCCAG
ATCATCTGCCAGCTCATCTGCCAGCTGACACACAGCCCATGGCTCCACTATTGCCAGTGCTGTAGCTGCACCAG

Figure S3. Site of the HBZ peak of enrichment. (**A**) The site of the HBZ peak of enrichment, denoted in red and labeled HBZ enhancer, and neighboring genes (ITGB1 and NRP1) are shown using the NCBI Genome Data Viewer (https://www.ncbi.nlm.nih.gov/genome/gdv/?org=homo-sapiens). (**B**) The chromosomal features in and around the peak of HBZ-enrichment were derived from ENCODE data [58] and are shown using the UCSC Genome Browser [58]. The vertical hatched lines show the boundaries of the peak of HBZ enrichment. AP-1 binding site predictions are shown as peaks in the density graph. (**C**) The DNA sequence corresponding to the HBZ peak encompasses bp 33,134,089-33,134,518 of chromosome 10 (GRCh38.p14 Primary Assembly). The bold sequences correspond to consensus AP-1 binding sites shown as the two peaks in panel B. Partial AP-1 binding sites are underlined.
